# Thermochemical Pretreatments of Organic Fraction of Municipal Solid Waste from a Mechanical-Biological Treatment Plant

**DOI:** 10.3390/ijms16023769

**Published:** 2015-02-09

**Authors:** Carlos José Álvarez-Gallego, Luis Alberto Fdez-Güelfo, María de los Ángeles Romero Aguilar, Luis Isidoro Romero García

**Affiliations:** 1 Department of Chemical Engineering and Food Technology, Faculty of Science, University of Cádiz, Puerto Real 11510, Cádiz, Spain; E-Mails: carlosjose.alvarez@uca.es (C.J.A.-G.); angeles.romero@uca.es (M.A.R.A.); 2 Department of Environmental Technologies, CASEM building, University of Cádiz, Puerto Real 11510, Cádiz, Spain; E-Mail: alberto.fdezguelfo@uca.es

**Keywords:** anaerobic, pretreatments, solubilisation, thermochemical

## Abstract

The organic fraction of municipal solid waste (OFMSW) usually contains high lignocellulosic and fatty fractions. These fractions are well-known to be a hard biodegradable substrate for biological treatments and its presence involves limitations on the performance of anaerobic processes. To avoid this, thermochemical pretreatments have been applied on the OFMSW coming from a full-scale mechanical-biological treatment (MBT) plant, in order to pre-hydrolyze the waste and improve the organic matter solubilisation. To study the solubilisation yield, the increments of soluble organic matter have been measured in terms of dissolved organic carbon (DOC), soluble chemical oxygen demand (sCOD), total volatile fatty acids (TVFA) and acidogenic substrate as carbon (ASC). The process variables analyzed were temperature, pressure and NaOH dosage. The levels of work for each variable were three: 160–180–200 °C, 3.5–5.0–6.5 bar and 2–3–4 g NaOH/L. In addition, the pretreatment time was also modified among 15 and 120 min. The best conditions for organic matter solubilisation were 160 °C, 3 g NaOH/L, 6.5 bar and 30 min, with yields in terms of DOC, sCOD, TVFA and ASC of 176%, 123%, 119% and 178% respectively. Thus, predictably the application of this pretreatment in these optimum conditions could improve the H_2_ production during the subsequent *Dark Fermentation* process.

## 1. Introduction

Anaerobic digestion is a widely applied technology for the treatment of organic wastes. This process is mainly developed through four main stages: hydrolysis, acidogenesis, acetogenesis and methanogenesis. Generally, hydrolysis is considered the rate-limiting step of anaerobic digestion of solid wastes [1]. Hence, the hydrolysis stage is decisive for the OFMSW biodegradation and it determines the overall rate of the process. Furthermore, if the hydrolysis stage is faster, the acidogenesis step (VFA and rich-H_2_ biogas) starts-up earlier and, hence, the global rate of the anaerobic digestion is higher [2]. It must be noted that nowadays, the H_2_ is considered the energetic vector of the future and, therefore, the increase of its generation in the acidogenic step by means of pretreatment may be an interesting target. To reach this goal, thermal, chemical (or combination of them) or biological pretreatments have been commonly developed in the literature as possible processes to accelerate the organic matter solubilisation process from organic wastes, as the sludge coming from wastewater treatments plants (WWTP), or complex wastes with small particle size [3,4,5,6]. The organic matter solubilisation is referred to the process in which the non-solubilized organic matter (organic matter contained inside particles for example) is solubilized to the liquid phase through the pretreatments discussed above.

About the thermal pretreatments, it must be highlighted that they have been successfully scaled to industrial scale [7]. Furthermore, the application of high temperatures allows the removal of pathogenic microorganisms and improves the properties of the digestate as its dehydration due to a decrease in its viscosity [8]. Most of the scientific references are focused on improving the performance of anaerobic digestion of solid waste, such as sludge from WWTP and lignocellulosic waste, by applying thermal pretreatments using temperatures between 50 and 250 °C. In this regard, it should be noted that very little research has been developed to improve the anaerobic digestion of OFMSW.

Analogously, most of the studies developed with chemical pretreatments were applied to sewage sludge and lignocellulosic waste [9]. Among the limited information regarding the application of chemical pretreatments to OFMSW are the results obtained by Lopez-Torres and Llorens. These authors reported improvements of 11.5% in the methane productivity by applying alkaline agents [10]. Furthermore, Neves *et al.* [11] reported improvements of 100% by applying NaOH as alkaline agent in concentrations of 0.3 g/gTS pretreating barley waste, and Patil *et al.* [12] reported the implementation of this type of pretreatment on waste such as water hyacinth with low lignocellulose content. The results of their study showed that, if the waste has low lignin content, this type of pretreatment (acid or alkaline) has little effect compared to mechanical pretreatments.

Finally, with respect to biological pretreatments, these may be developed in aerobic or anaerobic conditions and through the application of specific enzymes (such as peptidase, lipase and carbohydrolase) to the anaerobic reactors to pre-hydrolyze the waste. Similarly to the thermal and chemical pretreatments, biological pretreatments have been little studied to improve the anaerobic digestion of OFMSW. Most published works are focused on sewage sludge from WWTP and pulp and paper industries. It has been reported that the application of precomposting as aerobic biological pretreatment improves the hydrolysis of the hardly biodegradable solid waste due to the high concentration of hydrolytic enzymes that are provided by the biological agent, mature compost in this case [13].

However, the literature about the applications of pretreatments on organic wastes processed in full-scale plants and with high particle size, as the OFMSW coming from a full-scale mechanical-biological treatment (MBT) plant used in this study, is very limited. In fact, few references have been found about this topic [1,14,15,16] in which the best organic matter solubilisation was achieved by means of thermochemical pretreatments at 180 °C, 5 bar and 3 g NaOH/L as alkaline agent.

About the NaOH dosage, Kim *et al.* [17] tested different alkaline agents and the best solubilisation yield (SY), 85% expressed in terms of COD, was obtained applying a dose of 7 g NaOH/L. On the other hand, Penaud *et al.* [18] reported 5 g NaOH/L as the optimum concentration to reach the maximum organic matter solubilisation.

In the above mentioned papers [1,14,15,16], the range of the operational conditions tested must be considered as preliminary since there was not specific literature about the applications of pretreatments on OMFSW coming from a full-scale MBT plant. Therefore and based on the previous information, the main goals of this paper are three:

(1) To determine with more accuracy, (while reducing the operational variables ranges), the best conditions for improving the organic matter solubilisation according to preliminary results obtained by Fdez-Güelfo *et al.* [1] in order to provide further detailed information that will complement the conclusions obtained by these authors.
(2) To study the effect of the operation time on the SY. In the literature, most thermochemical pretreatments are only developed at 30 min of operation time, with optimum value generally for pretreatments of sludge from WWTP [1,16,17,18,19,20,21,22,23,24].
(3) To contrast the SY estimated in terms of classical parameters of organic matter (DOC, sCOD and TVFA) with a new indirect parameter defined by Fdez-Güelfo *et al.* [25], the “*acidogenic substrate as carbon (ASC)*”, in order to evaluate the possible effect of the pretreatments on the H_2_ production during the acidogenic phase of a sequential anaerobic process.

## 2. Results and Discussions

In order to determine the best conditions to develop the thermochemical pretreatments among those tested in this study, it is necessary to analyze the effect of each variable (temperature, pressure, NaOH dosage and operation time) individually taking into account the SY calculated according to Equation (1) (see Experimental section below).

### 2.1. Optimizing the Operational Variables

**Figure 1:**
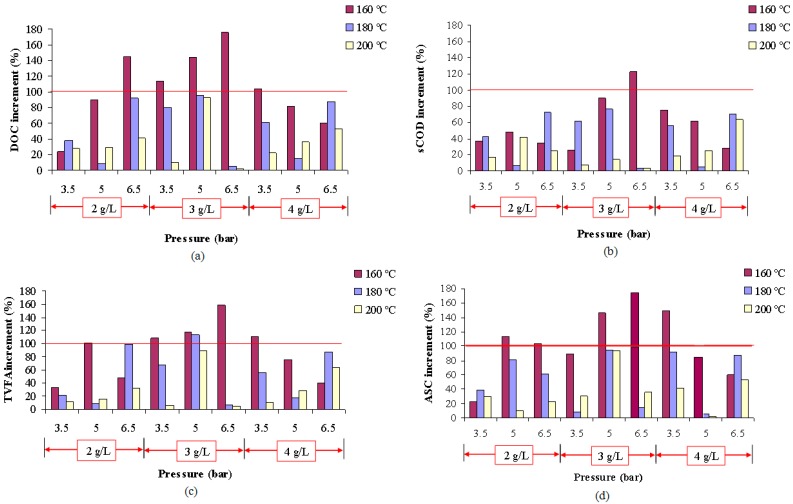
Effect of the temperature on the SY, expressed in terms of DOC (**a**); sCOD (**b**); TVFA (**c**) and ASC (**d**), at different conditions and 30-min operation time.

With regard to the temperature, according to the SY presented in Figure 1, it can be stated that the most successful thermochemical pretreatment has been generally performed at the lower temperature among those tested, 160 °C.

This result is in accordance with the results obtained by Delgenés *et al.* [26] who reported that temperatures ranging between 90 and 160 °C cause an increase in the organic matter solubilisation. For temperatures higher than 200 °C, the SY is considerably lower. It seems to be linked to the maintainence of high temperature during long operation periods, which may induce losses by thermal decomposition, polymerization or even caramelization processes of an important fraction of organic matter solubilized to the liquid phase and, therefore, the final increments of the pretreatment on the organic matter solubilisation could decrease. 

Fdez-Güelfo *et al.* [1] reported that the most efficient temperature was 180 °C when the temperatures tested were 120, 150 and 180 °C but it is was not possible to fence the optimum. In this sense, the present work has increased the accuracy of the results reported by these authors, since the optimum temperature, in which SY measured as DOC, sCOD, TVFA and ASC is higher than 100%, has been found at 160 °C and therefore it could be restricted between 150 and 180 °C.

**Figure 2:**
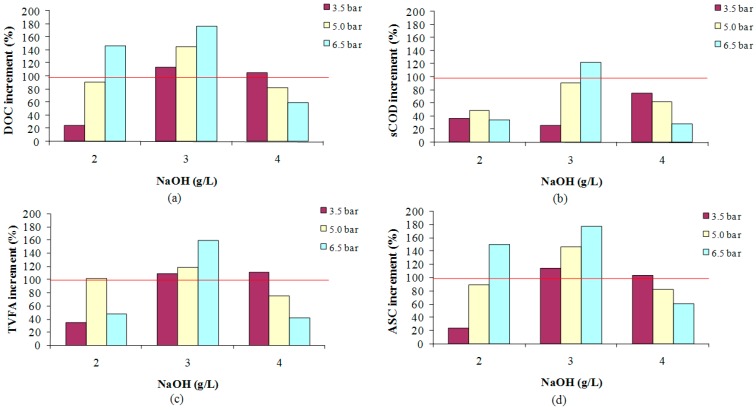
Effect of the NaOH additions on the SY, expressed in terms of DOC (**a**); sCOD (**b**); TVFA (**c**) and ASC (**d**), at 160 °C and 30-min operation time.

In regard to the influence of the NaOH additions, as it can be seen in Figure 2, at a temperature of 160 °C the optimum NaOH concentration is generally the intermediate between 2 and 4 g/L (3 g/L) for the different pressures tested, *i.e.*, the higher NaOH concentration is not required to reach the maximum SY. In this sense, it is very important to highlight that the best SY has been obtained at lower temperature (160 °C). The above results may be associated to the fact that the hydrolytic capacity of the NaOH decreases when the operational temperature is very high. In fact, several authors have observed a decrease in this synergic effect between NaOH and temperatures over 180 °C [26]. This fact may be due to high temperature enhancing the formation of refractory compounds which are difficult to solubilize. In addition, at these conditions some intermolecular reactions occur between solubilized and non-solubilized compounds that lead to the formation of complex substances.

In addition, this result is in accordance with the conclusion obtained by Fdez-Güelfo *et al.* [1]. These authors reported that the most efficient NaOH concentration was 3 g/L when the tested NaOH concentrations were 1, 3 and 5 g/L. A narrower range (2–4 g/L) for the optimum had been established in this study.

**Figure 3:**
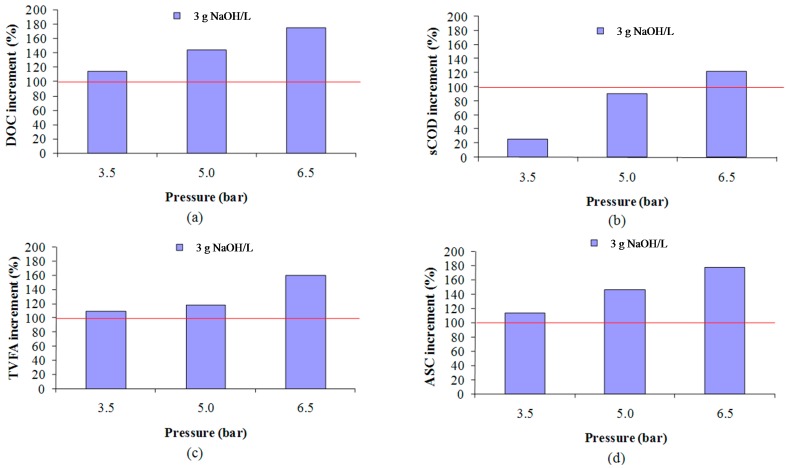
Effect of the pressure on the SY, expressed in terms of DOC (**a**); sCOD (**b**); TVFA (**c**) and ASC (**d**), at 160 °C, 3 g NaOH/L and 30-min operation time.

With respect to the influence of the pressure, according to Figure 3, at 160 °C and 3 g NaOH/L, the best SY among those tested was obtained for 6.5 bar of pressure. If this result is compared with the conclusions reported by Fdez-Güelfo *et al.* [1], which found that the most efficient pressure was 5 bar when the tested pressures were 1, 5 and 10 bar, this study indicates that the optimum pressure must be placed between 5 and 10 bar. Again, this work has increased the accuracy of the results reported by these authors, but further studies must be faced to check if the optimum is higher or lower than 6.5 bar. Thus, as it can be seen in Figure 1, high pressures appear to have positive effects on the SY when the NaOH additions are low (2 to 3 g/L) while it is negative when the NaOH additions are higher (4 g/L).

Finally, with regard to the influence of the operation time on the SY, after selecting the optimum conditions (160 °C, 6.5 bar and 3 g NaOH/L), three additional operation times (15, 60 and 120 min) were tested in order to determine the effect of this variable on the SY. The results obtained are shown in Table 1.

**Table 1:** Solubilisation yield (SY), expressed in terms of DOC, sCOD, TVFA and ASC, at 160 °C, 6.5 bar and 3 g NaOH/L, for the different operation times.

Operation Time (min)	Solubilisation Yield (%)			
	DOC	sCOD	TVFA	ASC
15	107.2	52.2	63.08	97.4
30	175.51	122.74	118.90	177.95
60	156.7	114.7	89.1	161.3
120	34.9	62.8	19.0	31.4

As can be seen, the optimum operation time for this work is 30 min, the same as for sewage sludge and complex wastes with small particle size [1,17,18,19]. In fact, according to these results it seems to be linked to long operation periods (60–120 min) which may induce the effects of Maillard reaction (caramelization processes previously described) generally reported by several authors whose studies were focused on thermal pretreatments applied to solid waste such as the OFMSW, municipal biomass waste, barley waste or food waste [7,8,9,10,11,12,13].

### 2.2. Expected Effect of Pretreatment on the Extent of Acidogenesis and H_2_ Production

ASC is the fraction of solubilized organic matter that has not been transformed into VFA and therefore, this indirect parameter may be used to study the behavior of the acidogenesis phase [25]. Thus, ASC may be used to describe how this pretreatment may affect the acidogenic phase of a *Dark Fermentation* process and, therefore, on the H_2_ production. If the solubilisation yield in terms of ASC is high, higher amounts of organic matter could be transformed to VFA during the acidogenesis step and, therefore, higher H_2_ production could be expected during a sequential anaerobic digestion process of pretreated waste.

As can be seen in Table 1, when the optimum conditions are applied (160 °C, 6.5 bar, 3 g NaOH/L and 30 min of operation time) the maximum increment of ASC (178%) is achieved. Therefore, it may be expected that implementation of this thermochemical pretreatment under these optimum conditions could promote the VFA and H_2_ generation during the acidogenic phase in a subsequent anaerobic process.

### 2.3. Statistical Analysis

A statistical study of the data was developed by means of the statistical software IBM^®^ SPSS^®^ Statistics version 19.0. Firstly, *Tukey* test (significant difference) was applied to determine if the experimental data have a normal distribution [27]. Values of the significance (*S*) higher than 0.05 imply that experimental data have a normal distribution. For all variables, the *Tukey* test backs ANOVA analysis.

Secondly, an analysis of variance (One-Way ANOVA) was carried out in order to estimate if each factor has a significant effect on the analyzed parameters. Similarly, following the analysis of variance, if the significance value is less than 0.05 indicates that there are significant differences among the data. As it can be seen in Table 2, the temperature is the only factor that unequivocally shows a significant effect on the solubilisation yield when the organic matter is expressed in terms of DOC, TVFA and ASC. Statistical results for pressure and NaOH dosage are not unequivocally conclusive.

**Table 2:** ANOVA analysis: influence of factors (temperature, pressure and NaOH dosage) on each parameter.

Parameter	Temperature	Pressure	NaOH Dosage
**∆DOC**	**0.003**	0.543	0.340
**∆sCOD**	0.077	0.801	0.761
**∆TVFA**	**0.010**	0.685	0.292
**∆ASC**	**0.003**	0.543	0.349

Bold values indicate that factor affects significantly (*S* < 0.05).

Finally, a specific statistical analysis of temperature factor by *Tukey* tests allows corroboration that there are significant differences among the levels (160, 180 and 200 °C) tested in this study (Table 3). Thus, if 160 °C is the temperature in which the solubilisation yields measured experimentally are generally higher (Figure 1), it may be concluded that this level of temperature is the best from the experimental and statistical point of view.

**Table 3:** Tukey test: multiple comparisons among levels of temperature factor.

Temperatures (ºC)		∆DOC	∆sCOD	∆TVFA	∆ASC
**160**	**180**	**0.028**	0.573	0.096	**0.028**
	**200**	**0.003**	0.064	**0.009**	**0.003**
**180**	**160**	**0.028**	0.573	0.096	**0.028**
	**200**	0.575	0.365	0.498	0.586
**200**	**160**	**0.003**	0.064	**0.009**	**0.003**
	**180**	0.575	0.365	0.498	0.586

Bold values indicate that factor affects significantly (*S* < 0.05).

## 3. Experimental Section

### 3.1. Methodology

Thermochemical pretreatments assays were carried out in inert atmosphere (N_2_). The employed waste was OFMSW coming from a 15-mm screen (trommel) of a full-scale 880 t/day MBT plant (named *Las Calandrias)* placed in Jerez de la Frontera (Cadiz, Spain). The following operative variables were studied: temperature, pressure and sodium hydroxide (NaOH) dosage as alkaline agent. In addition, different operation times were tested in this work in order to study its possible effect on the organic matter solubilisation.

The average particle size of the waste was lower than 15 mm and its characterization is shown in Table 4. It must be highlighted that the morphology of the waste used in the experiments was not altered in any manner, *i.e.*, the waste was not processed through drying or milling. In addition, the composition of the OFMSW (44% organic matter) was not altered in the laboratory and, therefore, it had the complete, original features of real waste.

**Table 4:** Physico-chemical characterization of the organic fraction of municipal solid waste.

Parameter	Value
pH	7.23
Density (g/L)	0.79
Alkalinity (g·CaCO_3_/L)	6.36
Ammonia (g·NH_4_^+^–N/L)	0.019
Total solids (%)	0.598
Volatile solids (%)	0.265
Dissolved organic carbon (mg·C/g sample)	26
Total volatile fatty acids (mg·AcH/g sample)	10.03

Sample units had been expressed at wet basis.

### 3.2. Design of Experiments

The experiment was conducted according to a 3^n^-type factorial design (3 being the number of the variable and “n” being the number of levels of each variable tested). The variables were temperature, pressure and alkaline reagent dosage, as described in Table 5. The factorial design established 27 experiments.

**Table 5:** Design of experiments.

Variable	Ranges Tested by Fdez-Güelfo *et al.* [1]	Optimum Values (Fdez-Güelfo *et al.* [1])	Ranges Tested in this Study
Temperature (°C)	120–150–180	180	160–180–200
Pressure (bar)	1–5–10	5	3.5–5.0–6.5
^(^*^)^ Dosage (g NaOH/L)	1–3–5	3	2–3–4

^(^*^)^ It is the amount of NaOH (in grams) applied per liter of diluted OFMSW with a TS concentration of 20%.

The assays were carried out in a 1-L non-stirred pressure vessel (*Parr*™, series 4600–4620) equipped with two proportional-integral-derivative (PID) controllers for temperature and pressure fitting. The pressure was regulated by means of a pneumatic over-pressure valve activated by a compressor. A muffla heating jacket was also employed to heat the system.

The reactor was filled till 75% of its capacity with OFMSW for each test. The waste was diluted by means of the addition of tap water until it reached a total solids (TS) concentration of 20%. The alkaline reagent was added from a 10 M-NaOH solution.

It is very important to highlight that in this study, (unlike the methodology followed by Fdez-Güelfo *et al.* [1], in which the operation time was set just on 30 min), four different operation times (15, 30, 60 and 120 min) were tested, from the optimization of the temperature, pressure and dosage variables, in order to study the possible effect of this variable on the solubilisation yield.

### 3.3. Determining the Solubilisation Yield (SY)

The effect of the pretreatments on the organic matter solubilisation has been measured in terms of dissolved organic carbon (DOC), total volatile fatty acids (TVFA), soluble chemical oxygen demand (sCOD) and acidogenic substrate as carbon (ASC).

Solubilisation yields, in terms of increments of DOC, TVFA, sCOD in the liquid phase, were calculated as shown in Equation (1):

(1) $$SY\left(\%\right)=\;\;\left(\frac{SOM_{F}-SOM_{I}}{SOM_{I}}\right)\cdot 100$$

Where SOM_F_ and SOM_I_ are the final and initial soluble organic matter respectively expressed as DOC, TVFA, sCOD or ASC, the last one calculated indirectly from classical parameters Equations (2) and (3) according to Fdez-Güelfo *et al.* [25].

(2) ASC [ML3]=DOC−DAC

(3) DAC [ML3]= ∑i= 2i=7[AiH· ni·12MWi]

where:

■ DAC, the dissolved acid carbon and it represents an average of carbon considering the “*carbon/molecular weight*” ratios of each VFA independently measured by gas chromatography.
■ DOC [M/L^3^], the dissolved organic carbon measured by carbon analyzer.
■ A_i_H [M/L^3^], represents the concentration of every individual VFA measured by gas chromatography.
■ *n*_i_, the number of carbons of A_i_H.
■ MW_i_, the molecular weight of A_i_H.

In this study, the pretreatment has been considered successful when the organic matter solubilisation yield is higher than 100%, maximum value of efficiency obtained by other authors [17,18,19].

### 3.4. Analytical Techniques

TVFA, sCOD and DOC were measured in supernatant after a lixiviation step. Lixiviation was performed in a flask with continuous stirring by adding 10 g of sample to 100 mL deionized water for 20 min, according to Romero-Aguilar *et al.* [28]. Individual VFA (from C2 to C7, including iC4, iC5 and iC6) levels were determined by gas chromatography (SHIMADZU GC-17 A) with a flame ionization detector (FID) and a capillary column filled with Nukol (polyethylene glycol modified by nitro-terephthalic acid). The temperatures of the injection port and detector were 200 and 250 °C, respectively. Helium was the carrier gas at 50 mL·min^−1^. In addition, nitrogen gas was used as make up at 30 mL·min^−1^ flow rate. On the other hand, DOC was measured according to 5310B standard method [29] in a combustion-infrared carbon analyzer (*Shimadzu TOC5050A*). Finally, sCOD was determined according to the colorimetric standard method 5220C [29].

## 4. Conclusions

Based on the above discussion, the following conclusions may be stated:

(1) With respect to the influence of the factors temperature, pressure and NaOH dosage on the SY, it can be concluded that the best results have been obtained at 160 °C, 3 g NaOH/L, 6.5 bar and 30 min of operation time. In these conditions, the solubilisation yield obtained in terms of DOC, sCOD, TVFA and ASC were 176%, 123%, 119% and 178% respectively. Hence, the optimum conditions reported by Fdez-Güelfo *et al.* [1], 180 °C, 3 g NaOH/L and 5 bar, may be redefined with higher accuracy in order to improve the efficiency of this pretreatment for applying it to OFMSW coming from full-scale MBT plants.
(2) According to the statistical analysis, the temperature is the only factor that unequivocally shows a significant effect on the solubilisation yield when the organic matter is expressed in terms of DOC, TVFA and ASC.
